# Effect of Sintering Process on Ionic Conductivity of Li_7-x_La_3_Zr_2-x_Nb_x_O_12_ (x = 0, 0.2, 0.4, 0.6) Solid Electrolytes

**DOI:** 10.3390/ma14071671

**Published:** 2021-03-29

**Authors:** Lei Ni, Zhigang Wu, Chuyi Zhang

**Affiliations:** School of Materials Science and Engineering, Chang’an University, Xi’an 710064, China; 2018231027@chd.edu.cn (Z.W.); 2019231001@chd.edu.cn (C.Z.)

**Keywords:** Li_7_La_3_Zr_2_O_12_, solid electrolytes, Nb doping, sintering process, ionic conductivity

## Abstract

Garnet-type Li_7_La_3_Zr_2_O_12_ (LLZO) is considered as a promising solid electrolyte. Nb-doped LLZO ceramics exhibit significantly improved ion conductivity. However, how to prepare the Nb-doped LLZO ceramics in a simple and economical way, meanwhile to investigate the relationship between process conditions and properties in Li_7-x_La_3_Zr_2-x_Nb_x_O_12_ ceramics, is particularly important. In this study, Li_7-x_La_3_Zr_2-x_Nb_x_O_12_ (LLZN_x_O, x = 0, 0.2, 0.4, 0.6) ceramics were prepared by conventional solid-state reaction. The effect of sintering process on the structure, microstructure, and ionic conductivity of LLZN_x_O (x = 0, 0.2, 0.4, 0.6) ceramics was investigated. Due to the more contractive Nb-O bonds in LLZN_x_O ceramics, the cubic structures are much easier to form and stabilize, which could induce the decreased preparation time. High-performance garnet LLZN_x_O ceramics can be obtained by optimizing the sintering process with lower calcining temperature and shorter holding time. The garnet samples with x = 0.4 calcined at 850 °C for 10 h and sintered at 1250 °C for 4 h exhibit the highest ionic conductivity of 3.86 × 10^−4^ S·cm^−1^ at room temperature and an activation energy of 0.32 eV, which can be correlated to the highest relative density of 96.1%, and good crystallinity of the grains.

## 1. Introduction

As a typical representative in the solid electrolyte family (including perovskite-type, NASICON-like, LI-SICON-like, sulfide-based series and garnet-type), garnet-type Li_7_La_3_Zr_2_O_12_ (LLZO) is considered as a promising material that could solve the safety issues of the leakage of flammable organic liquid electrolytes in traditional Li-ion batteries because of its high Li-ion conductivity, high stability versus metallic Li, wide electrochemical window, and good chemical stability [1,2,3,4]. LLZO has two crystalline structures, cubic phase and tetragonal phase. Although the cubic LLZO shows higher Li-ion conductivity (10^−4^~10^−3^ S·cm^−1^) than the tetragonal one, it is unstable at room temperature [5] and its conductivity is still lower than that of commercially used liquid organic electrolytes. Moreover, cubic LLZO is very difficult to synthesize, as preparation by conventional solid-state reaction requires a high sintering temperature and quite long holding time (about 1200 °C and more than 10 h).

Lots of work has been devoted to stabilizing the cubic structure and enhancing the Li-ion conductivity of LLZO ceramics by ion substitution, such as partial substitution of Li ions by Ge, Al, Fe, and Ga [6,7,8,9] and of Zr ions by Ta, Nb, and Y et al. [10,11,12,13,14,15]. Besides the type and content of substitution ions, the structure and conductivity of LLZO-based ceramics are quite sensitive to the preparation process. The conductivity of Ta-doped LLZO ranged from 10^−6^ to 10^−4^ S·cm^−1^ with different sintering processes [16]. These phenomena were also observed in Nb-doped LLZO ceramics [17,18,19,20,21].

Since Ohta et al. [17] first reported the enhanced Li-ion conductivity in Li_6.75_La_3_Zr_1.75_Nb_0.25_O_12_ ceramics prepared by solid state reaction at 1200 °C for 36 h, many methods have been applied to optimize the preparation process, such as sol-gel, co-precipitation, and hot pressing et al. [18,19,20,21]. However, these methods are relatively complicated with low efficiency and high cost. Thus, how to prepare the cubic LLZO solid electrolytes in a simple and economical way, meanwhile to investigate the relationship between process conditions and properties of Li_7-x_La_3_Zr_2-x_Nb_x_O_12_ ceramics, is particularly important. In this study, Li_7−x_La_3_Zr_2−x_Nb_x_O_12_ (LLZN_x_O) (x = 0, 0.2, 0.4, 0.6) ceramics were prepared by conventional solid-state reaction. The effect of processing conditions as well as the content of doping ions on the structure and ionic conductivity of LLZN_x_O (x = 0, 0.2, 0.4, 0.6) ceramics were systematically investigated.

## 2. Materials and Methods

LLZN_x_O (x = 0, 0.2, 0.4, 0.6) ceramics were synthesized by conventional solid-state reaction using the raw materials of Li_2_CO_3_ (99.99%), La_2_O_3_ (99.99%, preheated at 900 °C for 10 h), ZrO_2_ (99.99%) and Nb_2_O_5_ (99.99%). Stoichiometric amounts of raw materials were mixed by ball-milling with zirconia balls for 10 h in isopropanol at 300 rpm. A 10 wt% excess of Li_2_CO_3_ was added to compensate for the loss of lithium during the calcination process. Dried powders were calcined in an alumina crucible at 800, 850, and 900 °C for 10 h, respectively. The calcined powders were further ball-milled for 10 h to obtain pristine powders. Then the pristine powders were pressed into green pellets of 12 mm in diameter under 100 MPa. Green pellets covered by pristine powders were sintered around 1250 °C for a different time.

The crystalline structure was evaluated by X-ray diffraction (XRD, Bruker D8 Advance) with Cu Kα radiation (λ = 1.5148 Å). The microstructure was characterized by scanning electron microscope (SEM, FEI, Helios NanoLab G3 UC). The relative density values of ceramic samples were obtained by the Archimedes method with the equation as *ρ* = *m*_1_ *ρ*_w_/( *m*_1_−*m*_2_). where *m*_1_ is the mass of the sample in the air, *m*_2_ is the mass of the sample in the absolute ethyl alcohol, *ρ*_w_ is the density of the absolute ethyl alcohol. The ionic conductivity of ceramics with Au-sputtered electrodes were measured using the AC impedance spectroscopy (Wayne Kerr 6500B) in the frequency range of 20 Hz to 120 MHz and temperature range of 25 to 50 °C; The average thickness of the samples is about 1.05 mm.

## 3. Results and Discussion

**Figure 1:**
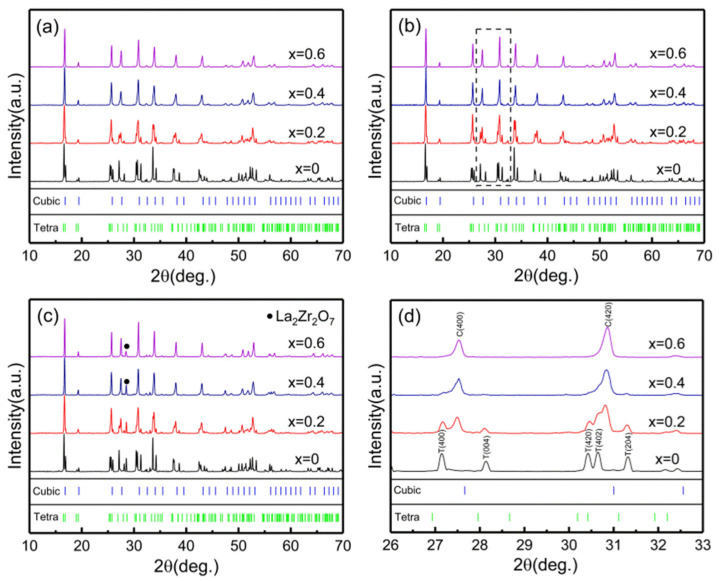
XRD patterns of LLZN_x_O (x = 0, 0.2, 0.4, 0.6) powder calcined at different temperatures for 10h. (**a**) 800 °C, (**b**) 850 °C, (**c**) 900 °C. (**d**) Enlarged XRD patterns of LLZN_x_O (x = 0, 0.2, 0.4, 0.6) powder calcined at 850 °C for 10 h from 26 to 33°.

Figure 1 shows the XRD patterns of LLZN_x_O (x = 0, 0.2, 0.4, 0.6) powder calcined at 800, 850, and 900 °C, respectively, for 10 h. Nb-doped samples with x = 0.4, 0.6 calcined at 800 °C show the cubic garnet structure, while the specimen with x = 0.2 shows a cubic garnet structure mixed with few tetragonal phases. The diffraction peaks of all the samples calcined at 850 °C become significantly sharper, indicating better crystallinity. A small amount of second phase (La_2_Zr_2_O_7_) is observed in all LLZN_x_O powders calcined at 900 °C. Therefore, the calcining temperature of 850 °C was selected. As shown in Figure 1d, the diffraction peaks of pure LLZO powder belong to a tetragonal phase, while the structure of LLZN_x_O (x = 0.2, 0.4, 0.6) powder obviously changes from tetragonal to perfect cubic phase with increasing Nb content. The substitution of Nb on Zr sites can easily stabilize the cubic structure of LLZO ceramics. 

**Figure 2:**
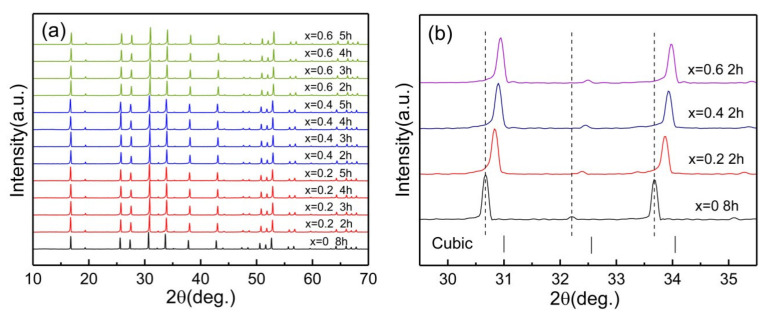
(**a**) XRD patterns of LLZN_x_O (x = 0, 0.2, 0.4, 0.6) ceramics sintered at 1250 °C for different times. (**b**) The enlarged XRD patterns of LLZN_x_O (x = 0, 0.2, 0.4, 0.6) ceramics sintered at 1250 °C for 2 h from 29.5 to 35.5°.

LLZN_x_O (x = 0, 0.2, 0.4, 0.6) ceramics have a narrow sintering temperature range. Dense samples can only be well sintered at 1250 °C. Figure 2a shows the XRD patterns of LLZN_x_O (x = 0, 0.2, 0.4, 0.6) ceramics sintered at 1250 °C for different times. The diffraction peaks of all ceramic samples were identified as cubic garnet structures without impurity phases. The holding time of pure LLZO samples can be reduced to 8 h, while the Nb-doped samples can be obtained with shorter time (less than 5 h). As shown in Figure 2b, the diffraction peaks of LLZN_x_O (x = 0, 0.2, 0.4, 0.6) ceramics show large angle migration with increasing Nb content, which should be attributed to the smaller-radius ions of Nb^5+^ (0.69 Å) than those of Zr^4+^ (0.72 Å). In Nb-doped LLZN_x_O ceramics, the attraction between Nb and O ions is much stronger than that between Zr and O ions. Due to the contraction of Nb-O bonds, the cubic structures are much easier to form and stabilize. Therefore, the easier it is to form the cubic phase, the shorter the time of the sintering process will be.

**Figure 3:**
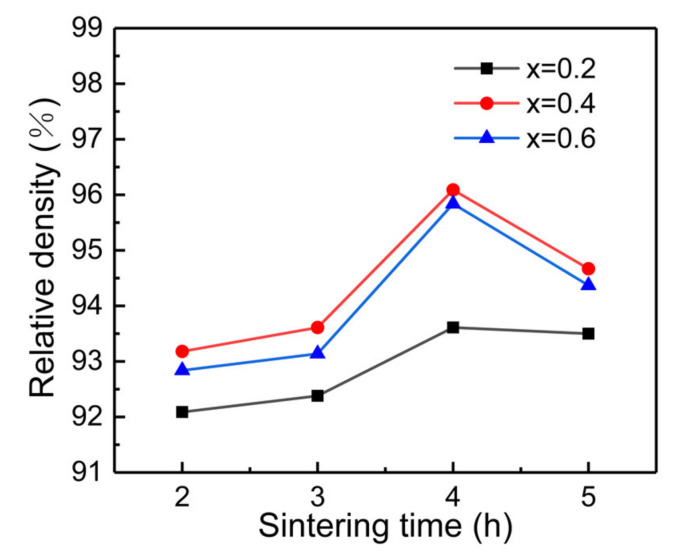
Relative density of LLZN_x_O (x = 0.2, 0.4, 0.6) ceramics.

The relative density of LLZN_x_O (x = 0.2, 0.4, 0.6) ceramics is shown in Figure 3. Compared with pure LLZO ceramics (relative density is 89.2%), the relative density of Nb-doped LLZO ceramics is improved (all above 92%), which first increases and then decreases with increasing holding time. The evaporation of Li during long-term sintering may result in the decrease of relative density. Meanwhile, the relative density shows the similar regularity with increasing Nb content. The highest relative density (96.1%) is obtained in the samples with x = 0.4 sintered at 1250 °C for 4 h.

**Figure 4:**
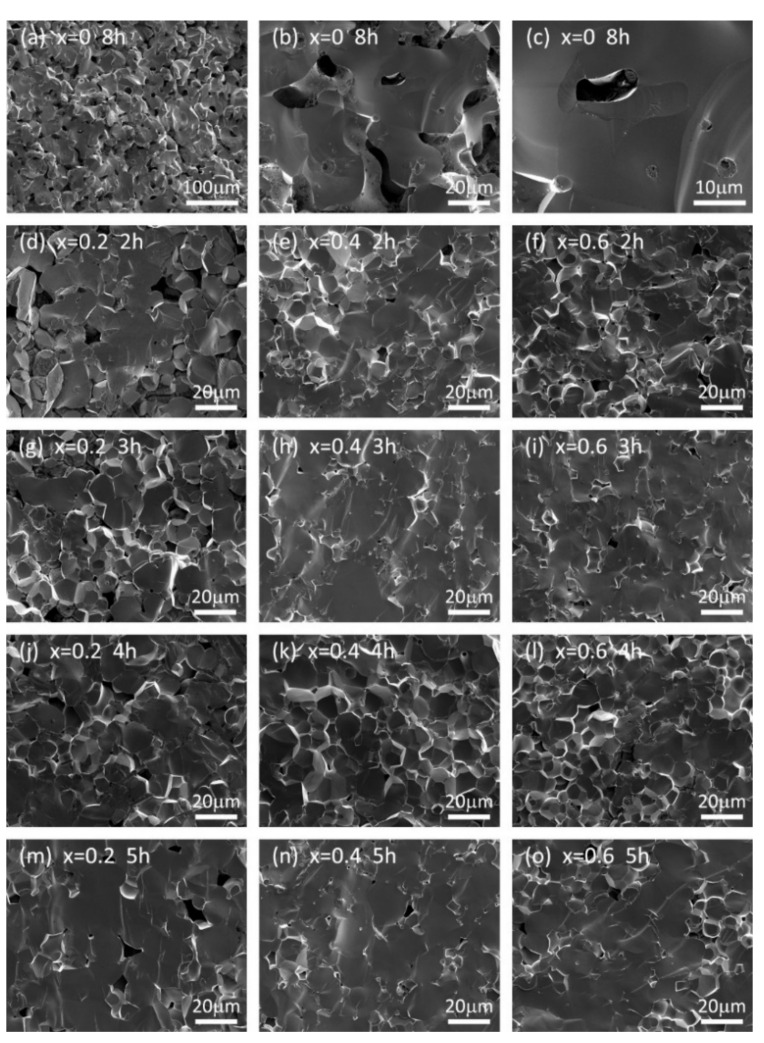
Cross-section SEM images of LLZN_x_O (x = 0, 0.2, 0.4, 0.6) ceramics sintered at 1250 °C for different holding times. (**a**–**c**): x = 0, 8 h at different magnification. (**d**–**f**): x = 0.2, 0.4, 0.6 sintered for 2 h respectively. (**g**–**i**): x = 0.2, 0.4, 0.6 sintered for 3 h respectively. (**j**–**l**): x = 0.2, 0.4, 0.6 sintered for 4 h respectively. (**m**–**o**): x = 0.2, 0.4, 0.6 sintered for 5 h respectively.

Figure 4 shows the cross-section SEM images of LLZN_x_O (x = 0, 0.2, 0.4, 0.6) ceramics sintered at 1250 °C for different holding times. As shown in Figure 4a–c, the grain size of pure LLZO ceramics is inhomogeneous, and ranges from 20 to 50 μm. There are numerous pores in pure LLZO ceramics. With increasing Nb content, LLZN_x_O ceramics exhibit much smaller grain size, but good crystallinity and connections between the grains. The porosity in LLZN_x_O ceramics decreases significantly with the increase in holding time from 2 to 4 h, and then increases when the holding time increases to 5 h, which could be attributed to more Li volatilization in the samples during the longer-time and high-temperature sintering process. 

**Figure 5:**
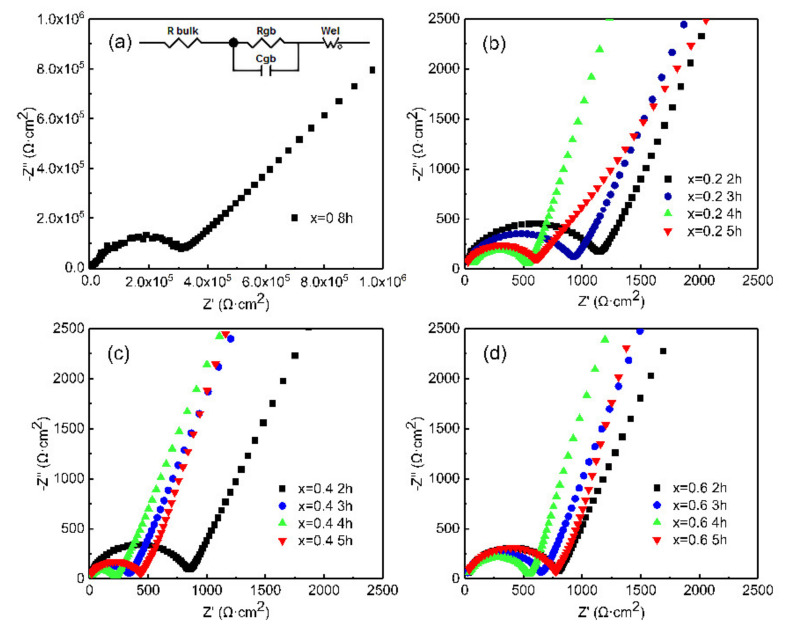
Nyquist plots of LLZN_x_O (x = 0, 0.2, 0.4, 0.6) ceramics at room temperature. (**a**) x = 0, (**b**) x = 0.2, (**c**) x = 0.4, (**d**) x = 0.6. *R*_bulk_, *R*_gb_(*C*_gb_), and *W*_el_ in equivalent circuit are resistance of grain, resistance (capacitance) of grain boundary and Warburg impedance of electrode, respectively.

Nyquist plots of LLZN_x_O (x = 0, 0.2, 0.4, 0.6) ceramics at room temperature are shown in Figure 5. The Nyquist plots of all ceramic samples are composed of a semicircle in the high-frequency region and a tail in the low-frequency region. The semicircle in the high-frequency region is related to the total impedance of the grains and grain boundaries, while the tail in the low-frequency region is caused by the electrode effect. The total ionic conductivity of LLZN_x_O ceramics is calculated by the following Equation (1):

(1) $$\sigma =\frac{l}{RS}$$

where *R* is the resistance, *l* is the thickness of the samples, and *S* is the area of the electrode. The ionic conductivity of the ceramics is shown in Table 1.

The doping of Nb in LLZO ceramics not only greatly shortens the holding time, but also improves the ionic conductivity (up to 10^−4^ S·cm^−1^), which is two orders of magnitude higher than that of the pure LLZO ceramics (1.09 × 10^−6^ S·cm^−1^). The increased ionic conductivity can be attributed to the decreased number of pores and denser structure of LLZN_x_O ceramics. The ionic conductivity of LLZN_x_O (x = 0.2 and 0.6) ceramics increases with increasing holding time from 2 to 5 h. The ionic conductivity of LLZN_x_O (x = 0.4) ceramics first increases from 2 h to 4 h and then decreases as the holding time increases to 5 h, which can be explained by the same trends in microstructure as well as the density. The samples with x = 0.4 sintered at 1250 °C for 4 h represent the highest ionic conductivity (3.86 × 10^−4^ S·cm^−1^), which can be attributed to the highest density and improved crystallinity.

**Figure 6:**
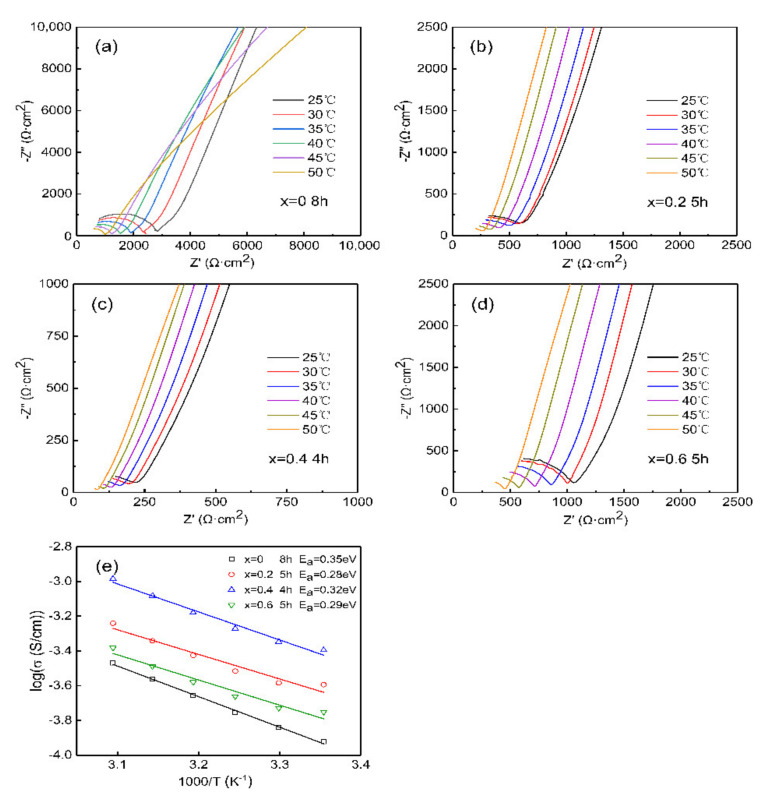
Nyquist plots of LLZN_x_O (x = 0, 0.2, 0.4, 0.6) ceramics at 25–50 °C: (**a**) x = 0, 8h (**b**) x = 0.2, 5 h, (**c**) x = 0.4, 4 h, (**d**) x = 0.6, 5 h. (**e**) Temperature dependence of conductivity of LLZN_x_O (x = 0, 0.2, 0.4, 0.6) ceramics. Solid symbols are experimental data, and the lines are fitting curves according to Arrhenius law.

Figure 6 shows the Nyquist plots of LLZN_x_O (x = 0, 0.2, 0.4, 0.6) ceramics at 25 °C–50 °C, where Figure 6b–d are the Nb-doped samples with the highest ion conductivity, respectively. As the temperature increases, the semicircle gradually decreases and the ionic conductivity increases. The temperature dependence of the ionic conductivity is shown in Figure 6e and can be expressed by the Arrhenius equation:

(2) $$\sigma =A\exp\left(\frac{-E_{a}}{K_{b}T}\right)$$

where *σ* is the ionic conductivity, *A* is the pre-exponential factor, *T* is the absolute temperature, *E_a_* is the activation energy and *K_b_* is the Boltzmann constant. The activation energy was calculated from the slope of log *σ* versus 1000/*T*, which is in the range of 0.28–0.35 eV. 

The heterovalent substitution of Zr^4+^ for Nb^5+^ could increase the Li^+^ vacancies concentration in order to provide the charge compensation. In parallel it leads to a decrease in the activation energy for ionic conductivity and facilitating the diffusion of Li^+^ ions. Besides the effect of ion substitution on the Li^+^ vacancies, the sintering process also plays an important role in the concentration of Li+ vacancies. With increasing sintering temperature and time, the volatilization of Li ions in LLZN_x_O ceramics is inevitable, which could induce more Li vacancies. When the holding time is short, the conductivity of LLZN_x_O ceramics could increase due to the generation of a small amount of Li+ vacancies. However, the conductivity of LLZN_x_O ceramics decreased with increasing the holding time to a certain value, which is probably attributed to worse density and abnormal grain growth caused by the generation of numerous of Li^+^ vacancies.

## 4. Conclusions

In this study, LLZN_x_O (x = 0, 0.2, 0.4, 0.6) solid electrolyte was prepared by conventional solid-state reaction. The effect of the sintering process on the structure, microstructure, and ionic conductivity of LLZN_x_O (x = 0, 0.2, 0.4, 0.6) solid electrolytes was investigated. High-performance garnet LLZN_x_O solid electrolyte can be prepared by optimizing the sintering process. Li_6.6_La_3_Zr_1.6_Nb_0.4_O_12_ (x = 0.4) ceramics sintered at 1250 °C for 4 h have the highest ionic conductivity of 3.86 × 10^−4^ S·cm^−1^ at room temperature and an activation energy of 0.32 eV, which can be attributed to the highest relative density of 96.1% and good crystallinity of the grains.

## Figures and Tables

**Table 1:** Total ionic conductivity of LLZN_x_O (x = 0, 0.2, 0.4, 0.6) ceramics at different sintering time.

Composition Sintering Time (h)	Conductivity (S·cm^−1^)	Composition Sintering Time (h)	Conductivity (S·cm^−1^)	Composition Sintering Time (h)	Conductivity (S·cm^−1^)
x = 0.2, 2 h	1.09 × 10^−4^	x = 0.4, 2 h	1.56 × 10^−4^	x = 0.6, 2 h	1.49 × 10^−4^
x = 0.2, 3 h	1.43 × 10^−4^	x = 0.4, 3 h	3.56 × 10^−4^	x = 0.6, 3 h	1.92 × 10^−4^
x = 0.2, 4 h	2.37 × 10^−4^	x = 0.4, 4 h	3.86 × 10^−4^	x = 0.6, 4 h	2.36 × 10^−4^
x = 0.2, 5 h	2.49 × 10^−4^	x = 0.4, 5 h	2.62 × 10^−4^	x = 0.6, 5 h	2.42 × 10^−4^

## Data Availability

The article includes all data.
